# Prospective Tracking of Donor-Reactive T-Cell Clones in the Circulation and Rejecting Human Kidney Allografts

**DOI:** 10.3389/fimmu.2021.750005

**Published:** 2021-10-14

**Authors:** Constantin Aschauer, Kira Jelencsics, Karin Hu, Andreas Heinzel, Mariella Gloria Gregorich, Julia Vetter, Susanne Schaller, Stephan M. Winkler, Johannes Weinberger, Lisabeth Pimenov, Guido A. Gualdoni, Michael Eder, Alexander Kainz, Anna Regina Troescher, Heinz Regele, Roman Reindl-Schwaighofer, Thomas Wekerle, Johannes Bernhard Huppa, Megan Sykes, Rainer Oberbauer

**Affiliations:** ^1^ Division of Nephrology and Dialysis, Department of Medicine III, Medical University of Vienna, Vienna, Austria; ^2^ Section for Clinical Biometrics, Center for Medical Statistics, Informatics and Intelligent Systems, Medical University of Vienna, Vienna, Austria; ^3^ Bioinformatics Research Group, University of Applied Sciences Upper Austria, Hagenberg im Muehlkreis, Austria; ^4^ Research Laboratory of Infection Biology, Department of Medicine, Medical University of Vienna, Vienna, Austria; ^5^ Department of Neuroimmunology, Medical University of Vienna, Vienna, Austria; ^6^ Department of Pathology, Medical University of Vienna, Vienna, Austria; ^7^ Department of General Surgery, Division of Transplantation, Section of Transplantation Immunology, Medical University of Vienna, Vienna, Austria; ^8^ Center for Pathophysiology, Infectiology and Immunology, Institute for Hygiene and Applied Immunology, Medical University of Vienna, Vienna, Austria; ^9^ Columbian Center for Translational Immunology, Department of Medicine, Columbia University, New York City, NY, United States

**Keywords:** T-cell receptor, alloreactivity, kidney transplant, rejection, next generation sequencing, network analysis

## Abstract

**Background:**

Antigen recognition of allo-peptides and HLA molecules leads to the activation of donor-reactive T-cells following transplantation, potentially causing T-cell-mediated rejection (TCMR). Sequencing of the T-cell receptor (TCR) repertoire can be used to track the donor-reactive repertoire in blood and tissue of patients after kidney transplantation.

**Methods/Design:**

In this prospective cohort study, 117 non-sensitized kidney transplant recipients with anti-CD25 induction were included. Peripheral mononuclear cells (PBMCs) were sampled pre-transplant and at the time of protocol or indication biopsies together with graft tissue. Next-generation sequencing (NGS) of the CDR3 region of the TCRbeta chain was performed after donor stimulation in mixed lymphocyte reactions to define the donor-reactive TCR repertoire. Blood and tissue of six patients experiencing a TCMR and six patients without rejection on protocol biopsies were interrogated for these TCRs. To elucidate common features of T-cell clonotypes, a network analysis of the TCR repertoires was performed.

**Results:**

After transplantation, the frequency of circulating donor-reactive CD4 T-cells increased significantly from 0.86 ± 0.40% to 2.06 ± 0.40% of all CD4 cells (p < 0.001, mean dif.: -1.197, CI: -1.802, -0.593). The number of circulating donor-reactive CD4 clonotypes increased from 0.72 ± 0.33% to 1.89 ± 0.33% (p < 0.001, mean dif.: -1.168, CI: -1.724, -0.612). No difference in the percentage of donor-reactive T-cells in the circulation at transplant biopsy was found between subjects experiencing a TCMR and the control group [p = 0.64 (CD4^+^), p = 0.52 (CD8^+^)]. Graft-infiltrating T-cells showed an up to six-fold increase of donor-reactive T-cell clonotypes compared to the blood at the same time (3.7 *vs.* 0.6% and 2.4 *vs.* 1.5%), but the infiltrating TCR repertoire was not reflected by the composition of the circulating TCR repertoire despite some overlap. Network analysis showed a distinct segregation of the donor-reactive repertoire with higher modularity than the overall TCR repertoire in the blood. These findings indicate an unchoreographed process of diverse T-cell clones directed against numerous non-self antigens found in the allograft.

**Conclusion:**

Donor-reactive T-cells are enriched in the kidney allograft during a TCMR episode, and dominant tissue clones are also found in the blood.

**Trial Registration:**

Clinicaltrials.gov: NCT: 03422224 (https://clinicaltrials.gov/ct2/show/NCT03422224).

## Background

Despite the improvement of immunosuppressive therapies in kidney transplantation, early acute T-cell-mediated rejection (TCMR) occurs in roughly 10% of patients and may impact graft function and survival if not detected early and treated appropriately (1–3). The pathophysiology of TCMR requires the interaction of the T-cell receptors (TCR) of CD4- and CD8-positive T-cells with an allo-peptide which is presented either in a self (indirect) or non-self (direct or semi-direct) human leukocyte antigen (HLA) molecule of antigen-presenting cells (4, 5).

This recognition of non-self peptides and HLA molecules leads to an alloresponse, and recently we showed that also mismatched non-HLA antigens between donor and recipients induce an immune response that is associated with a reduced graft survival (6).

In the setting of solid organ transplantation, recent papers described the clonality and diversity of the allo-TCR repertoire in the circulation after lymphodepletional induction therapy with anti-thymocyte globulin (ATG). DeWolf et al. showed that in healthy controls, roughly only 0.4 to maximal 10% of the whole TCR repertoire in blood is donor-reactive (7).

Accordingly, even in severe viral infections such as BK, CMV, or also tuberculosis, investigators showed that only a small fraction of each individual TCR repertoire undergoes viral or pathogen-specific expansion, but the majority of the repertoire remains unchanged (8–10). In the setting of BK viral clearance, Stervbo and colleagues showed how the TCR diversity and exhaustion state of BK-specific T-cells affect viral clearance and how the clonality and diversity is different to allograft rejection (11, 12).

In a previous study involving a small number of kidney transplant recipients, those receiving conventional immuno-suppression demonstrated a similar increase in circulating donor-reactive T cells defined by mixed lymphocyte reaction (MLR) sequencing, regardless of whether or not rejection occurred (13). What remains still unknown however is whether donor-reactive T-cell clones in the circulation mirror the infiltrating TCR repertoire in episodes of kidney transplant rejections (14). We previously addressed a similar question in the autoimmune setting of native kidney disease and one terminally failing allograft nephrectomy (15). We found that an individual’s T-cell and B-cell receptor repertoire in the kidney was different from the repertoire present in blood. However, 94% of the expanded clones in the kidney could also be detected in blood, although not all equally abundant. It is unknown, however, whether the alloresponse to a solid organ transplant is comparable to the findings in the autoimmune setting of native kidney disease and if dynamics in the allograft are reflected by a change of clonotypes in the circulation.

Morris and colleagues recently tracked donor-reactive T-cells in a cohort of four simultaneous kidney and bone marrow transplant recipients. Three of these patients developed operational tolerance, and TCR sequencing of peripheral T-cells suggested that long-term tolerance reflected clonal deletion of donor-reactive T-cells. The one patient with TCMR following immunosuppression withdrawal did not show evidence of deletion, in contrast to tolerant patients (13).

In a recent study focusing on the B-cell repertoire after transplantation, a difference in clonal expansion between patients with rejection and no rejection and the usage of specific immunoglobulin gene segments in those patients was shown (16). Clusters and pathogen-specific T-cells have been defined for the TCR repertoire using these approaches after infection or vaccination but have never been applied in the setting of kidney transplant recipients (8, 9, 17, 18).

Given the uncertainty of TCR repertoire kinetics in TCMR, we sought to prospectively elucidate the dynamics of clonality and diversity in blood and tissue of patients after kidney transplantation.

## Methods/Design

### Study Design

The detailed design of this prospective observational cohort study may be found elsewhere (19). In brief, we enrolled all incident non-sensitized deceased or live donor kidney transplant recipients at our center between November 1, 2017, and September 30, 2019, with a follow-up for 1 year after transplantation or until December 10, 2019, and medical induction therapy was uniformly basiliximab (anti-CD25 antibody) (19). The study was registered in a public clinical trial database on February 5, 2018 (ClinicalTrials.gov NCT03422224), and funded by a peer-reviewed funding by the Scientific Funds of the Austrian National Bank-OeNb project number 17289. The funding body had no influence on the design, collection, analysis, and interpretation of data and writing of the manuscript.

### Subjects

**Figure 1:**
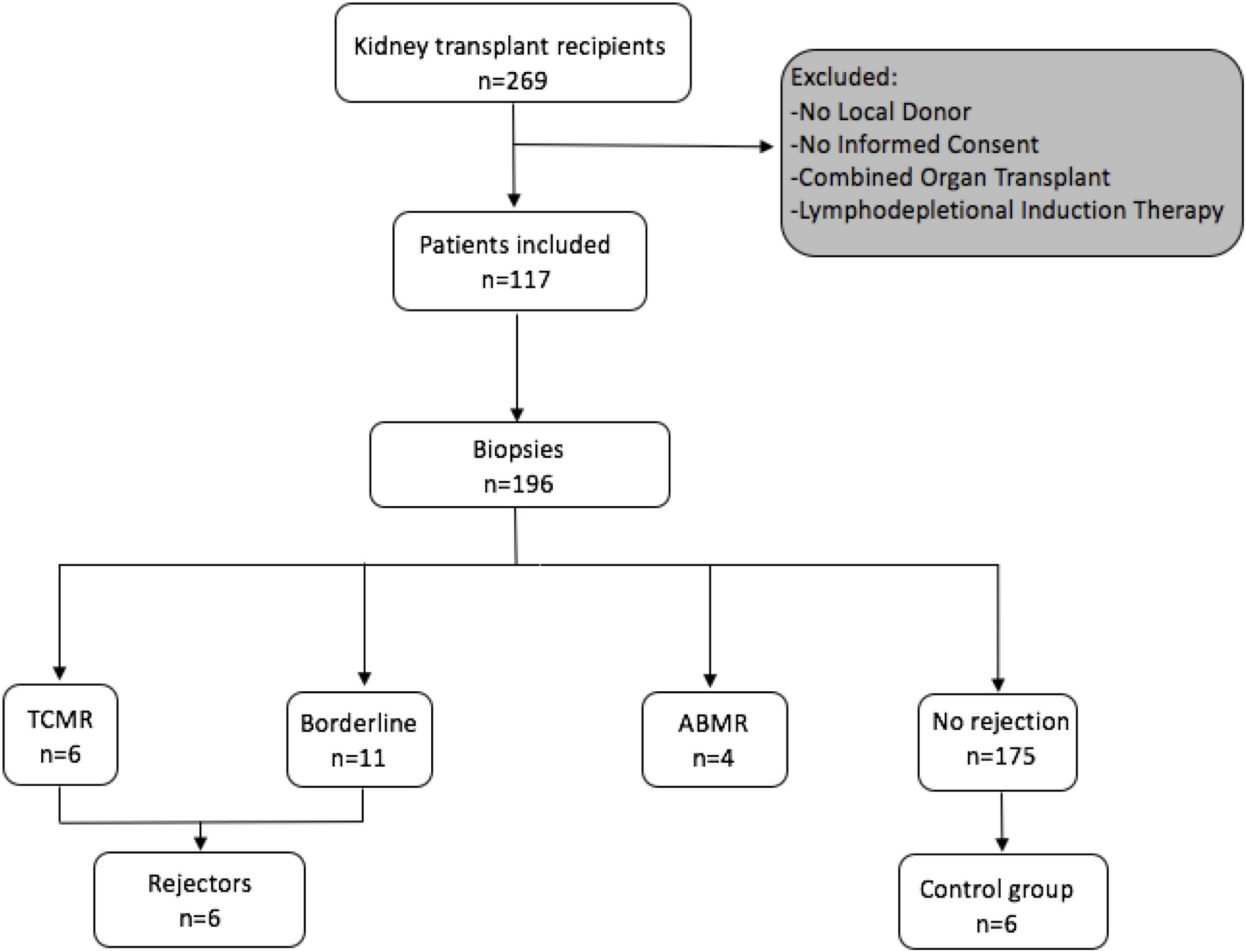
Flowchart following the STROBE statement of number of patients included prospectively and consecutive PBMC sampling and incidence of rejection episodes in the observed cohort.

A STROBE chart of included patients is provided in **Figure 1**. A total of 117 eligible recipients were included prior to transplantation, and recipient and donor peripheral mononuclear cells (PBMCs) were isolated at the time of transplantation and cryopreserved until analysis. As per center policy, all patients were invited for surveillance biopsies at 3 and 12 months, for-cause biopsies were obtained when indicated. We obtained 196 biopsies, of which 6 exhibited a TCMR, 4 an antibody-mediated rejection (ABMR), and 11 a borderline rejection according to the BANFF 2017 classification (20).

The blood samples of all patients experiencing a TCMR were processed for further *in vitro* experiments, but one had to be excluded due to insufficient T-cell numbers found in the pre-transplant blood and two were lost due to sorting errors. Overall, the MLR, FACS sorting, and sequencing were successfully performed for three patients with histologically proven pure acute TCMR and three patients with a borderline rejection. The TCRs were compared to a control group of six patients without histopathological signs of alloimmune response in kidney biopsies after transplantation. Biopsy specimens for TCR sequencing were available for two rejecting patients and compared to two biopsy specimens of the control group. The control group was selected based on the time of biopsy. Detailed patient characteristics are listed in **Table 1**. Wet-lab analyses were conducted according to standard protocols, and details were also described previously (19). In brief, after thawing of cryopreserved PBMCs, MLRs were performed by plating 2 × 10^5^ carboxyfluorescein succinimidyl ester (CFSE)-labeled (Invitrogen cat. #C34554) transplant-recipient cells and 2 × 10^5^ VPD-labeled (BD horizon cat. #562158) irradiated donor cells in each well of a 96-well plate. MLR cultures were incubated at 37°C for 6 days and were sorted on a FACS Aria II high-speed cell sorter for CD4^+^ CFSE^low^ and CD8^+^ CFSE^low^ T-cells (BD Pharmingen cat.# 552852, BioLegend cat.# 317426, BD Pharmingen cat.# 557834) (13, 21). Unstimulated pre- and post-transplant samples were sorted for CD4^+^ and CD8^+^ T-cells to define the bulk repertoire.

**Table 1:** Patient characteristics of selected individuals for TCR beta sequencing.

Subject	Gender	Cause of ESRD	MM- % PRA	Type of Tx	Graft dysfunction	Rejection	Timepoint of biopsy	Immunosuppressive therapy
R 14	Male	Unknown	1–2–1	DKD	N	N	POD 141	IL-2RA induction
			0%					+Steroid. MMF. Tac
R 148	Male	Unknown	0–1–2	DKD	N	N	POD 105	IL-2RA induction
			0%					+Steroid. MMF. Tac
R 190	Male	ADPKD	1–1–1	DKD	N	N	POD 81	IL-2RA induction
			0%					+Steroid. MMF. Tac
R 24	Male	ADPKD	1–1–0	LKD	N	N	POD 6	IAS (7x)+PA (1x)+ IL-2RA
			0%–ABOi					+Steroid. MMF. Tac
R 30	Male	Amyloidosis	1–1–1	LKD	Y	N	POD 41	IL-2RA induction
			0%					+Steroid. MMF. Tac
R 32	Female	Agenesis & Reflux NP	1–0–1	DKD	Y	N	POD 6	IL-2RA induction
			0%					+Steroid. MMF. Tac
R 106	Male	Unknown	1–2–1	DKD	Y	Y/N: Borderline	POD 7	IL-2RA induction
			0%					+Steroid. MMF. Tac
R 132	Female	IgA Nephritis	1–2–2	DKD	Y/DGF	Y: BANFF IIa	POD 7	IL-2RA induction
			0%					+Steroid. MMF. Tac
R 172	Male	ADPKD	0–1–1	DKD	Y	Y/N: Borderline	POD 62	IL-2RA induction
			0%					+Steroid. MMF. Tac
R 34	Male	Immune complex GN	1–0–1	DKD	Y	Y/N: Borderline	POD 126	IL-2RA induction
			0%					+Steroid. MMF. Tac
R 42	Male	Unknown	0–1–2	LKD	N	Y: BANFF Ib	POD 251	IAS (5x)+ IL-2RA
			0%–ABOi					+Steroid. MMF. Tac
R 43	Male	ADPKD	1–1–2	LKD	N	Y: BANFF IIb	POD 251	IL-2RA induction
			0%					+Steroid. MMF. Tac

POD, postoperative day; IAS, immunoadsorption; MMF, mycophenolat mofetil; MM, HLA mismatch (A-B-DR); DKD, diseased kidney donation; LKD, living kidney donation.

A historical cohort of seven kidney transplant recipients experiencing a cellular rejection episode was used to confirm findings in our cohort. Institutional ethics committee approval (EC NB: 1973/2017, signed 7/11/2017; EC NB.: 20303-0/2010-1018EKU (821/PI/010)) was obtained for all aspects of the study, and all study participants were included after signed informed consent prior to transplantation. Patient characteristics of the historical cohort are shown in **Supplementary Table 1**. The TCR sequencing method used for these samples has been described previously (15).

### TCR Repertoire Sequencing

RNA isolation, next-generation sequencing (NGS) library preparation, sequencing, and bioinformatics analysis were previously described (19). In brief, lymphocytes were sorted from PBMCs, and RNA isolation was done following the original TRIzol protocol (Invitrogen, Carlsbad, CA). RNA from allograft biopsies was isolated after cutting five histological slices of a biopsy core stored in Tissue-Plus OCT compound which were dissolved in TRIzol.

Total RNA was successfully isolated of all biopsy samples stored in Tissue-Plus OCT compound and subjected to library preparation except for one control due to mainly muscle and fat in the biopsy. For RNA from tissue specimens from the control group, no TCR amplicon could be obtained. This was concordant with histological absence of mononuclear cell infiltrates. TCR libraries were sequenced on an Illumina NextSeq 500, and reads were stored in separate fastq files per index. The MIGEC pipeline was used for further demultiplexing and unique molecular identifier (UMI)-guided consensus sequence assembly (22). Resulting consensus sequences were processed into clonotypes using MiXCR (23).

### Statistical Methods

#### Raw Data Processing

For each TCR β chain, the following information was retained for further analysis: CDR3 sequence (amino acid and nucleotide translation), cell counts, and frequency of clones with regard to the nucleotide sequence, V gene, D gene, and J gene. Ambiguous clonotypes with regard to its phenotype were assigned CD4 or CD8 according to the subset in which their maximum frequency across timepoints doubles the other. The remaining clonotypes were dropped due to their questionable affiliation. Clonotypes were defined as donor-reactive if they fulfilled a fold change equal or greater than 5 with regard to abundance between the stimulated and the unstimulated pre-transplant sample. The datasets for this study can be found in the European Genome-Phenome Archive (EGA, ID: EGAD00001007695).

#### Statistical Analysis

To correct for possible bias due to differences in the number of captured T-cells, the assessment of the frequency distribution of donor-reactive clonotypes in each repertoire was performed based on the mean value from 1,000 estimates, each obtained from random downsampling of the respective sample to the total number of T-cells in the overall repertoire with the lowest read count (24). The averaged estimates of the absolute count, cumulative fraction, and percentage of donor-reactive clonotypes between the pre-transplant and post-transplant repertoires after downsampling were then compared by means of paired t-tests, assuming that its prerequisites hold. Given the overall small sample size, Student’s t-test was used to compare means among different time points or between the two outcome groups since it mostly results (given approximate validity of the assumptions) in Type I error rates close to the 5% nominal significance level even in extremely small samples (25). Continuous variables were presented as mean ± standard deviation (SD). Multiple-group comparisons were performed by analysis of variance (ANOVA).

#### Diversity Analysis

The diversity of TCR clones in circulation and tissue was quantified by R20 and clonality. R20 is defined as the fraction of the most frequent clonotypes within the repertoire which together make up 20% of the entire repertoire. Hence, a relatively small R20 value indicates immunodominance of a subset of clonotypes, whereas an inflated number points toward the lack thereof. Clonality (C) provides a measure of normalized diversity and is derived from Shannon’s entropy of a sample (*H_obs_*).


$$C=1-\frac{H_{obs}}{H_{\max}}$$

*H_max_* is the maximum entropy possible for the observed sample space. Clonality lies between [0,1], where 0 represents maximal diversity and 1 the opposite with only one unique clonotype present.

Power law slopes for quantifying TCR repertoire diversity were constructed following the approach proposed by DeWolf (7). Each repertoire was split into its bulk and high-frequency component by specifying the cutoff as the second smallest unique expanded clone in terms of frequency. The bivariate relationship between clonal size and frequency of the bulk component presumably follows a power law distribution. Thus, to determine its power (the exponent of the power law), the slope of the log-transformed clonal size and frequency was estimated by univariable linear regression for each repertoire. A relatively steeper absolute slope value would indicate greater abundance of distinct clonotypes in the repertoire sample.

Unpaired t-tests were used to investigate differences in


$$\Delta \text{R}20=:\Delta \text{R}20_{\text{PostTX}}-\Delta R20_{\mathrm{Pr}eTX}\;\text{and}\;\Delta C:=\Delta C_{PostTX}-\Delta C_{\mathrm{Pr}eTX}$$

between the control group and rejectors. Paired t-tests were used to analyze pre-transplant and post-transplant samples since study subjects are compared at two different time points (26, 27). The threshold for statistical significance not adjusted for multiple testing was set to a p-value cutoff of 0.05. Due to the exploratory nature of the statistical analysis, p-values were not adjusted for multiple testing.

All analyses and statistical tests were performed in R version 4.0.2 (R Foundation for Statistical Computing, Vienna, Austria).

#### Comparison of Repertoires

The similarity overlap of inter-sample repertoires was assessed by the Jensen–Shannon divergence (JSD) of the top 1,000 clones, a quantitative measure of divergence between two probability distributions (24).

The function is bounded by 0 and 1, where a JSD equal to 1 would represent two fully distinct clonal distributions and 0 identical distributions.

#### Network Analysis

TCR networks were constructed as undirected and unweighted graphs with nodes corresponding to unique CDR3 β amino acid sequences connected by edges if a similarity condition based on the overlap of amino acid is fulfilled. The selected measure of node similarity was the Levenshtein distance, a string metric quantifying the difference between two CDR3 β amino acid sequences by counting the number of edits (insertion, deletion, substitution) required to convert one sequence into the other (28–30). Non-productive clones identified by sequences with start or stop codons were excluded from the analysis. In addition, the first and last three amino acids were omitted to perform the similarity overlap solely based on the inner segment of the CDR3 β amino acid sequence (8, 9). Graph-theoretical features such as modularity and edge density were extracted from the immune networks based on the top 10,000 most frequent clonotypes in the immune repertoire linked in case of a Levenshtein distance equal to 1. Edge density is the ratio between existing edges and the total number of edges possible. Low edge density indicates a sparse network in terms of connections between nodes. Modularity describes the subdivision of the network in groups of nodes with higher internal edge density (cluster) compared to in-between cluster connectivity by quantifying the number of edges within the clusters minus the expected number of edges in an equivalent network with random edge placement (31). Hence, a large modularity indicates a denser connectivity within given clusters of the network than expected by chance, while low modularity is associated with heterogeneous connectivity patterns across clusters. As a measure of nodal centrality, the betweenness centrality defined as the proportion of shortest paths passing through a node was assessed (32). Network analysis was performed using the R package igraph (33).

## Results

### Defining the Overall and Donor-Reactive TCR Repertoire

**Figure 2:**
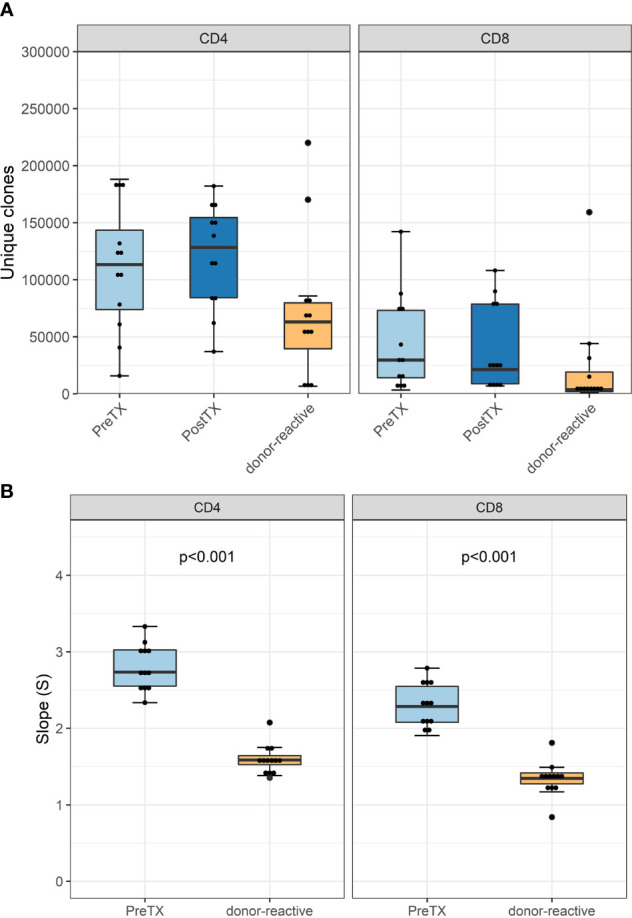
**(A)** Absolute number of unique clonotypes grouped by phenotype groups CD4 and CD8 for the pre-transplant and the donor-reactive repertoire samples. **(B)** Boxplot of the computed absolute values of the power law slopes grouped by phenotype groups CD4 and CD8 for the unstimulated and donor-reactive repertoire samples. This decrease in absolute values of the power law slope reflects a reduced diversity of the donor-reactive repertoire for CD4 and CD8 positive cells (n = 12). Power law slopes for every individual and timepoint are shown in **Supplementary Figures 1, 2**.

The numbers of detected TCR clonotypes pre-transplant were comparable to those after transplant (**Figure 2A**). As expected, responding cells from the MLR revealed a significantly smaller number of clonotypes compared to the unstimulated overall T-cells in the circulation. Detailed information on the number of clonotypes and clone numbers for each patient is shown in **Supplementary Tables 2–4**.

Diversity analysis using power law slopes confirmed the known features of donor-reactive T-cell repertoires; i.e., it represents a distinct population of T-cell clones, which is less diverse and with fewer unique clones than the unstimulated overall TCR repertoire (**Figure 2B**). The clonal abundance distribution of every subject pre- and post-transplant and donor-reactive repertoire are shown by power law slopes (**Supplementary Figures 1, 2**).

Analysis of VJ usage of each subject revealed a distinct VJ usage in the pre- and post-transplant samples for every individual as well as the individuals’ donor-reactive TCR repertoire without the domination of a specific V or J element for the overall donor-reactive repertoires (data not shown).

### Expansion of Donor-Reactive Clones in Circulation After Transplantation

**Figure 3:**
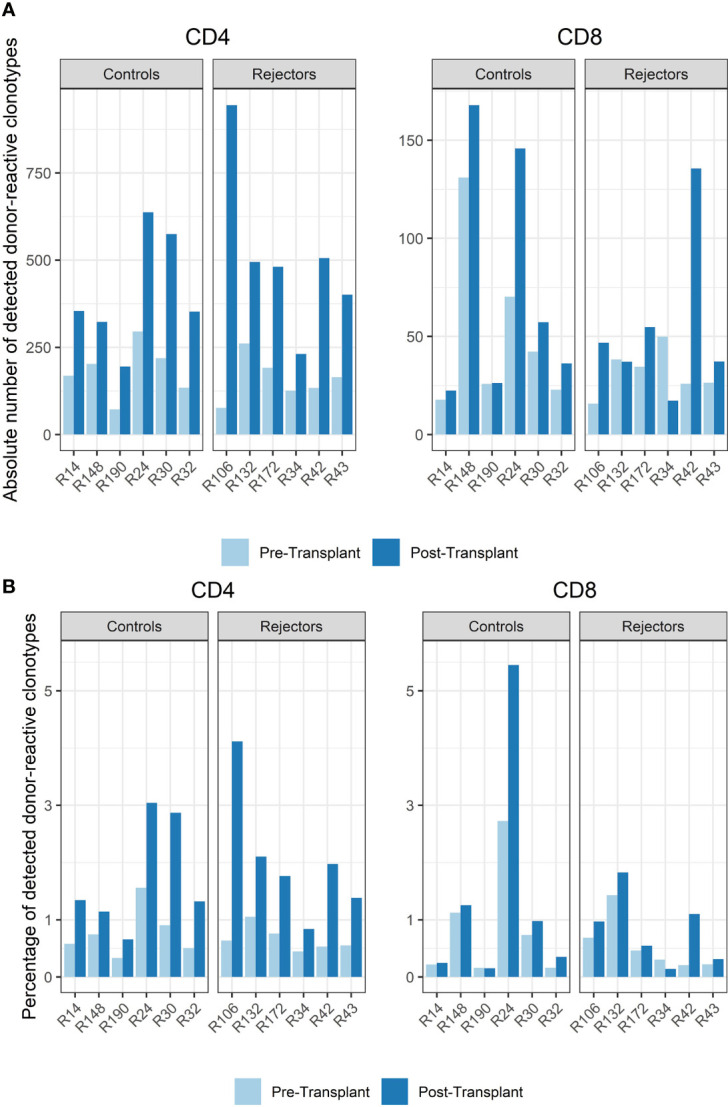
**(A)** Absolute number of detected donor-reactive clonotypes after normalization by downsampling to the smallest number of reads. **(B)** Percentage of detected donor-reactive clonotypes after normalization by downsampling to the smallest number of reads. A significant increase in the percentage of donor-reactive CD4-positive clonotypes (p < 0.001, mean dif.: -1.168, CI: -1.724, -0.612) and their absolute numbers (p < 0.001, mean dif.: -1.197, CI: -1.802, -0.593) was seen in patients with anti-CD-25 induction therapy after transplantation compared to pre-transplant assessed by paired and unpaired Student’s t-test.

Among all unique complementarity-determining region 3 (CDR 3) sequences found in the patients’ blood stream pre-transplant, the percentage of donor-reactive T-cell clonotypes ranged from 0.34 to 1.56% for CD4 and from 0.16% to 2.73% for CD8 clonotypes. Looking at the abundance of donor-reactive T-cells in the circulation, regarding their frequency, revealed that 0.43% to 1.92% of all CD4 and 0.15 to 1.49% of all CD8 cells were donor directed (**Figure 3**).

The timepoint of biopsy for the control *vs.* rejection group was 6 to 141 days (median 61 days) *vs.* 7 to 251 days (median 94 days) after transplantation, and the absolute number and percentage of donor-reactive clones in CD4 and CD8 T-cells increased in almost all subjects compared to pre-transplant in the peripheral blood stream (p < 0.001, mean dif.: -1.197, CI: -1.802, -0.593, **Figure 3**). This difference was also seen in the percentage of donor-reactive clonotypes at the timepoint of biopsy and also reached statistical significance for the CD4- but not the CD8-positive T-cells in rejecting patients and the control group (p < 0.001, mean dif.: -1.168, CI: -1.723, -0.612). These changes arose from donor-reactive clones with low frequencies and not detectable prior to transplantation without a predominance of certain clonotypes or increase of dominant single donor-reactive clones. The increase was detectable in all subjects including four individuals as close as 6 days after transplant and was also found in two other patients 251 days after transplant. No difference in the increase of donor-reactive T-cells was seen between rejectors and the control group [unpaired Student’s t test; p = 0.64 (CD4^+^) and p = 0.52 (CD8^+^)], and overall the increase in donor-reactive clones was more pronounced in the CD4 fraction compared to the CD8 alloclones.

### TCR Repertoire Diversity in the Circulation

**Figure 4:**
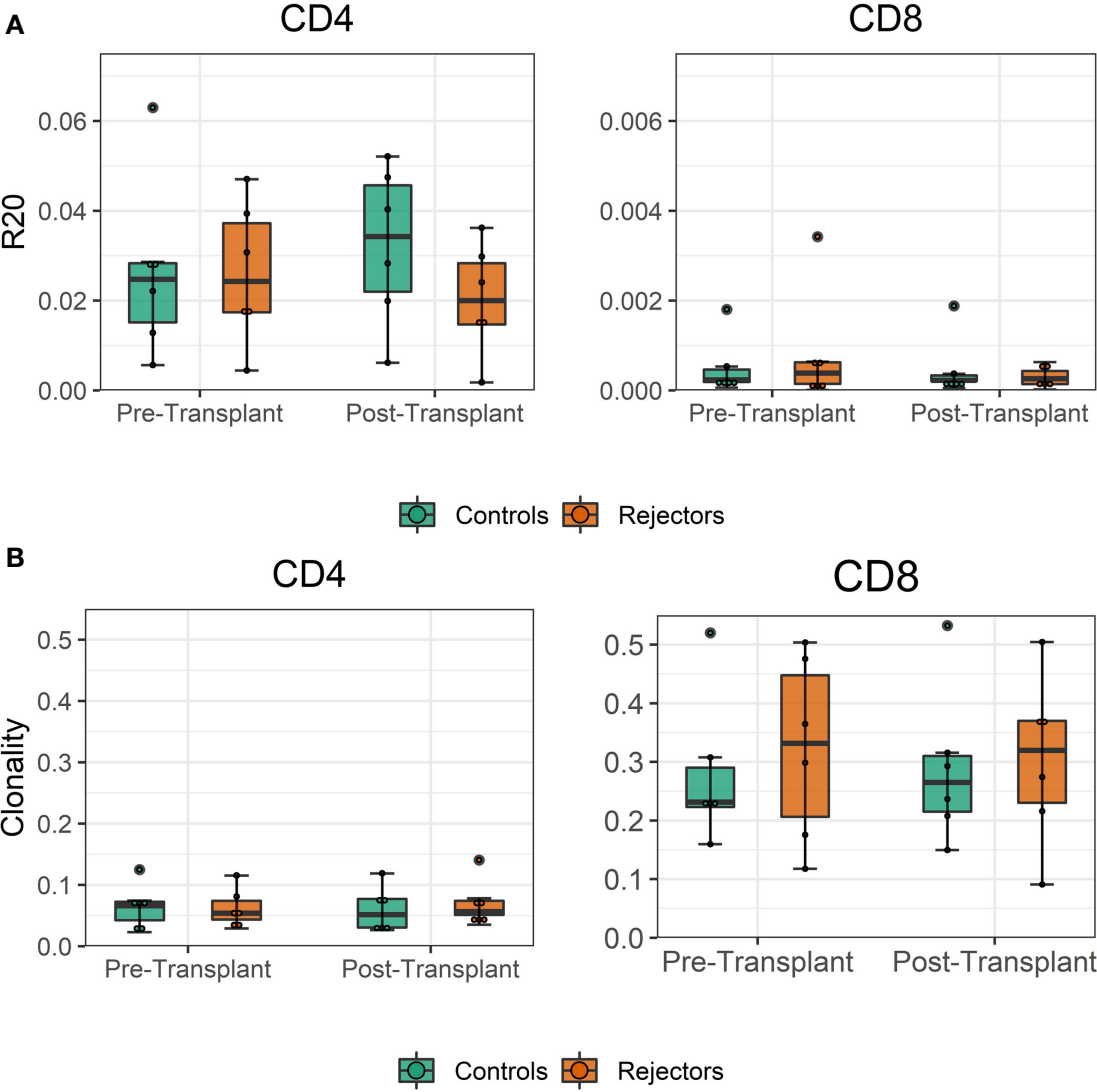
**(A)** Boxplot of clonality estimates of the pre- and post-transplant repertoire samples grouped by rejection. **(B)** Boxplot of R20 estimates of the clonotypes in the pre- and post-transplant repertoire samples grouped by rejection. No significant difference in diversity of the circulating TCR repertoire is detected between rejecting patients and control group assessed by two-sample Student’s t-tests (n = 12).

The diversity of pre-transplant repertoires measured by clonality ranged from 0.018 to 0.13 for CD4 and 0.13 to 0.5 for CD8 T-cells. The clonality of post-transplant samples showed comparable values to pre-transplant values, as shown in **Figure 4**. We did not find a difference in R20 as a metric for immune dominance comparing the rejecting and control groups (**Figure 4B**).

We quantified if the significant increase in alloclones was reflected by overall turnover in the TCR repertoire pre- and post-transplant, which would be indicated by a high Jensen–Shannon divergence (JSD). However, JSD values were low, indicating that there was little overall repertoire change in the sampling period and no statistical difference was seen in rejecting patients and the control group (**Supplementary Figure 3**).

We did not find a difference in the donor-specific repertoires in the peripheral blood of patients with rejection *vs.* control but an increase of these donor-reactive cells in all transplant recipients that was not accompanied by a significant change of the overall repertoire.

### Distinct Infiltration of Donor-Reactive T-Cells in the Kidney Graft at Rejection

In the biopsies of the two rejecting grafts, we found 6,186 and 23,108 T-cells comprising 3,248 and 10,266 different T-cell clonotypes in the samples with histological TCMR, and no TCR-specific RNA was detectable in the control biopsies. Out of these T-cells, the frequency of donor-reactive cells was 4.8% and 2.5% as defined by MLR pre-transplant, representing 3.7% and 2.4% of unique clonotypes, respectively. As FACS sorting was not feasible for these specimens, a separate analysis of CD4 and CD8 T-cells was not possible, and therefore further analysis was performed for the bulk repertoire.

**Figure 5:**
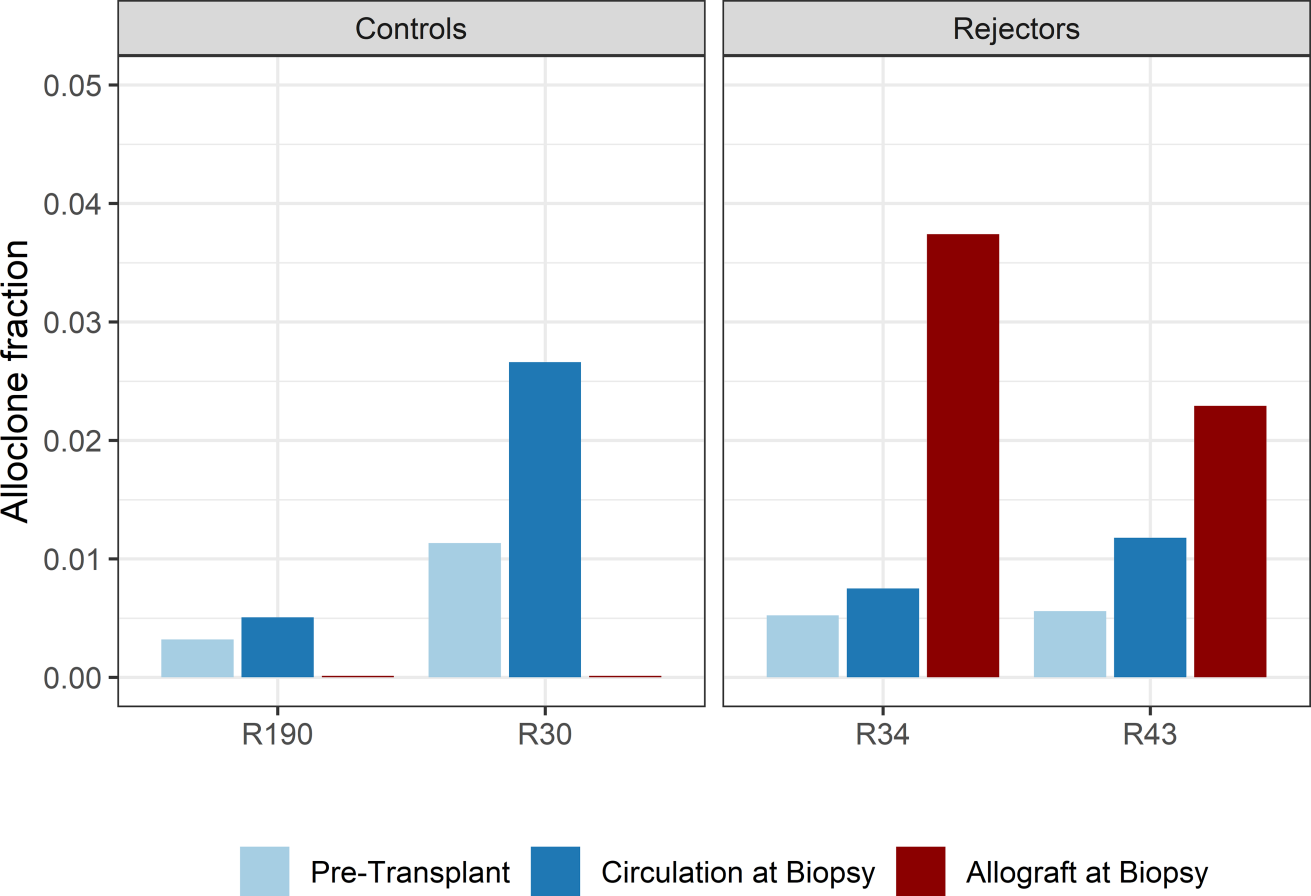
Bar plots of the fraction of detected donor-reactive clonotypes in the renal allograft after normalization by downsampling to the smallest number of reads. An increase in donor-reactive clones in the tissue (red bar) compared to pre-transplant (light blue) and post-transplant (dark blue) in the peripheral blood stream.

When compared to the fraction of donor-reactive T-cells in the circulation at the same time, an increase of donor-reactive clones in the allograft was found (**Figure 5**). As mentioned, the percentage of donor-reactive clonotypes in the biopsy was 3.7% and 2.4% compared to 0.6% and 1.5% in the peripheral blood at the same time for patient R34 and R43, respectively. Interestingly, a clonal dominance among those infiltrating T-cells was not seen, indicating a broad response of several antigen-specific T-cells during TCMR. R20 of the infiltrating TCR repertoire for R34 and R43 was 0.038 and 0.023, and clonality was 0.042 and 0.055, respectively. These values are comparable to the observed peripheral TCR repertoire (**Figure 4**). Contrary JSD values between the peripheral TCR repertoire at the time of biopsy and graft-infiltrating TCR was higher than the JSD values between the peripheral TCR repertoire pre-transplant and at the time of biopsy (**Table 2**). A list of the top 20 clonotypes present in the allograft including their frequency in the peripheral blood sample is found in **Supplementary Table 5**.

**Table 2:** Jensen–Shannon divergence (JSD) of the overall TCR repertoire considering only the top 1,000 clones of the pre-transplant (PreTX) and post-transplant (PostTX) samples.

Patient	Sample.1	Sample.2	JSD
R34	PreTX	Tissue	0.31
R34	PostTX	Tissue	0.32
R34	PreTX	PostTX	0.03
R43	PreTX	Tissue	0.21
R43	PostTX	Tissue	0.20
R43	PreTX	PostTX	0.03

The higher JSD values when comparing the peripheral blood pre- and post-transplant with the tissue sample imply an infiltration of distinct set of T-cells, different to the peripheral blood at the same time.

We also compared the VJ usage of allograft-infiltrating T-cells and circulating T-cells. Although we observed a stronger appearance of TRBV7-9xTRBJ2-7 and TRBV7-2xTRBJ2-7 usage in patient R34 and disappearance of TRBV7-2xTRBJ2-1 and TRBV7-9xTRBJ2-5 in patient R43 in the tissue, the overall VJ usage in periphery and tissue remained the same (**Supplementary Figure 4**). JSD for VJ usage between the peripheral blood and tissue was 0.065 and 0.063 for patient R34 and R43, respectively, and thus was very similar.

**Figure 6:**
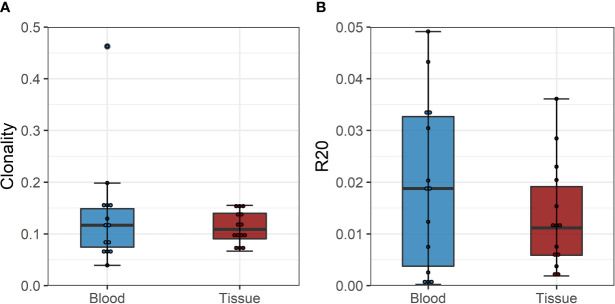
**(A)** Boxplot of clonality estimates of the circulating and graft infiltrating TCR repertoire in patients with a cellular rejection episode at timepoint of rejection. **(B)** Boxplot of R20 estimates of the clonotypes of the circulating and graft-infiltrating TCR repertoire in patients with a cellular rejection episode at timepoint of rejection. No significant difference in diversity of the circulating and graft-infiltrating TCR repertoire is detected (clonality: p=0.411, mean dif.: -0.023, CI: -0.036, -0.082; R20: p=0.117, mean dif.: 0.006, CI: -0.002, -0.014).

To confirm these findings, we performed a similar analysis on a historical cohort of seven biopsy and blood samples of kidney transplant recipients experiencing a cellular rejection episode. Despite the lack of a defined donor-reactive repertoire and a different sequencing approach, diversity analysis revealed similar results. There was no difference in diversity of the TCR repertoire measured by clonality and R20 between tissue sample and the circulation at the timepoint of biopsy. The mean clonality of the infiltrating compared to peripheral blood TCR repertoire was 0.112 *vs.* 0.135 (p = 0.411, mean dif.: -0.023, CI: -0.036, -0.082), and the R20 was 0.013 *vs.* 0.019 (p=0.117, mean dif.: 0.006, CI: -0.002, -0.014) (**Figure 6**). JSD for VJ usage between the peripheral blood and tissue ranged from 0.03 to 0.21 (**Supplementary Table 6**).

### Network Analysis of Overall and Donor-Reactive TCR Repertoire

**Figure 7:**
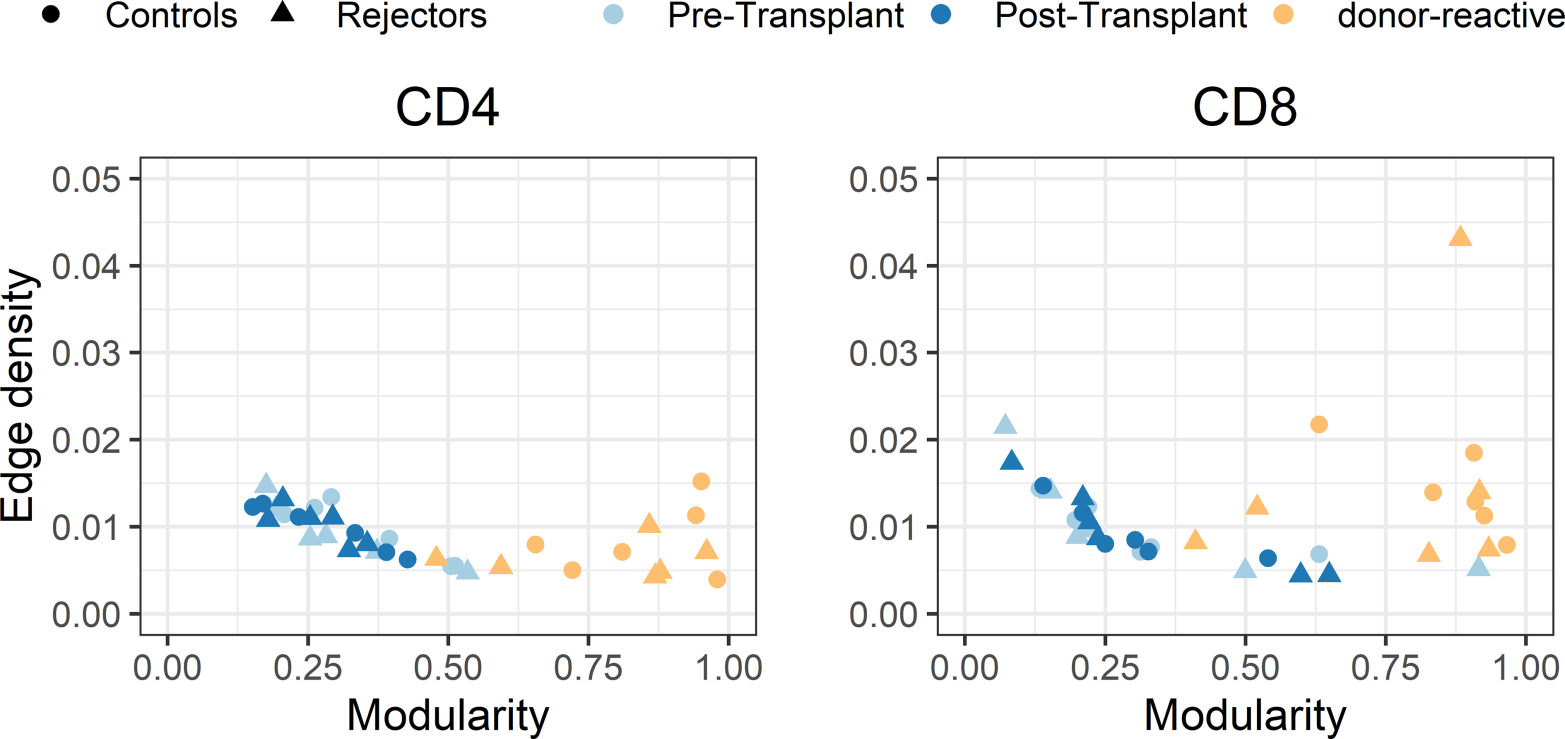
Scatterplot of edge density (%) and modularity of the individual-specific immune repertoire networks for CD4- and CD8-positive T-cells. The donor-reactive TCR repertoires (yellow) showed a significantly higher modularity assessed by paired Student’s t-test (p < 0.001) than the networks of the pre- and post-transplant bulk repertoires combined with comparable edge density across repertoires, suggesting a denser internal connectivity within the given clusters.

The individual-specific networks for the donor-reactive immune repertoires showed a significantly higher modularity than the networks of the pre-transplant (paired Student’s t test; p < 0.001; CD4 and CD8) and post-transplant repertoires (paired Student’s t-test; p < 0.001; CD4 and CD8), suggesting a closer connectivity within the formed clusters (**Figure 7**). In addition, there were no noticeable differences between the two timepoints or the donor-reactive repertoire in terms of edge density (ANOVA; p = 0.149 for CD4, p = 0.095 for CD8). This is a measure of overall connectivity in the network, which indicates that, although no observable increase in actual connections is measurable according to edge density across the different timepoints and the donor-reactive repertoire, the connections of the donor-reactive immune network are dominantly grouped in clusters (groups of densely connected nodes) which have sparse connections outside the cluster. In contrast, the low modularity values of the circulating overall TCR repertoire indicated heterogeneous connection patterns without the formation of highly connected topologic clusters. Taken together, this suggests a stronger subdivision of the donor-reactive immune networks into groups of clonotypes with similar CDR3 sequences. The outliers in the right panel of **Figure 7** are CD8 repertoires with low clonotype counts affecting the graph theoretical features.

In the individual-specific networks of the tissue repertoire, 9.3% and 22.5% of all donor-reactive clones found in the tissue were included in the respective individual-specific immune networks. Network analysis revealed that the most centrally connected clonotype in both of the individual-specific networks was donor-reactive determined by betweenness centrality (measure of node influence) and degree (measure of the connectivity between the nodes) (**Supplementary Figure 5**). These influential nodes were not clonotypes, which are dominant in terms of their frequency in the overall repertoire, but low-frequency clonotypes, which once more underpins that the bulk component of the repertoire exhibits CDR3 sequence similarity to donor-reactive clones rather than the high-frequency component.

These findings reflect that the alloreactive repertoire defined by the MLR is a distinct repertoire with stronger subdivision of the immune network into clusters which exhibit denser internal connections compared to the circulating overall TCR repertoire.

## Discussion

The main finding of our prospective cohort study was that pre-transplant defined donor-reactive T-cells infiltrated and accumulated in the transplant kidney at incidences of TCMR. The analysis of these infiltrating T-cells revealed that a distinct set of donor-reactive clones was found in the graft without the predominance of single clonotypes but more an infiltration of a broad number of different donor-reactive T-cells. By parallel sequencing of the TCR repertoire in the peripheral blood lymphocytes of these patients at the day of biopsy, we could show that there was little overlap in the repertoires of the two sites. We found an overall increase of donor-directed T-cells in the peripheral blood compared to pre-transplant but without detectable dynamics in those clones found in the allograft, reflecting distinct properties of these two sets at rejection.

The discrimination of patients experiencing a TCMR after a non-lymphodepletional induction therapy compared to recipients without signs of rejection in the kidney solely by analysis of the circulating donor-reactive repertoire was not possible, as a significant increase of donor-reactive T-cells was present in both groups.

The observation of a more marked expansion of donor-reactive TCRs in the rejecting kidney allograft than in the circulation at the same time is consistent with results obtained in intestinal transplant recipients, in which a remarkably high proportion of recipient TCRs infiltrating the allografts were shown to be donor-reactive during rejection episodes and to decline over time, as the rejection resolved (34). However, in the case of kidneys, prior studies showed that expanded immune cells in the peripheral blood were also expanded in diseased kidneys (15). However, the expansion of kidney-infiltrating T-cells was mostly seen in a non-transplant setting, where organ infiltration of T-cells is substantially different. Also, this study only analyzed one kidney allograft and this was already terminally failing, and it was not possible to draw conclusions on the donor-reactive TCR repertoire.

Other previous studies could partially demonstrate the presence and increase of donor-reactive T-cells in the circulation, but the role of these predefined T-cells during a TCMR episode and their presence or absence in the tissue were not proven in this setting (10, 14, 35).

Morris et al., introducing this novel technique to observe patients’ donor-reactive TCR repertoire in a combined bone marrow and kidney transplant setting, described differences in the circulation in individuals failing operational tolerance but were not able to analyze the tissue infiltrating repertoire (13).

Also, prior efforts by Alachkar et al. defined the TCR repertoire in the tissue of kidney transplant recipients but were not able to assess and distinguish between donor-reactive T-cells or just present T-cells in the allograft with unknown relevance (14). Similar to this study, Dziubianau et al. were able to perform TCR sequencing on kidney allografts and detect BK virus-specific T-cells, but the conclusion on the presence of donor-reactive T-cells was hampered as pre-transplant samples were missing for these patients (10).

Our data overcame this shortcoming as we define a bona fide donor-reactive TCR repertoire by an MLR with samples collected prior to transplant. These donor-reactive cells have shown in previous studies to represent the biological significant clones and stay comparable over time. We determined the TCR in blood at the same time as the TCMR was diagnosed by biopsy allowing to decipher the significance of these cells at rejection, although theoretically a certain number of T-cells could not have been sampled in the peripheral blood because they are sequestered in secondary lymphoid organs (7, 13).

A limitation of our paper is the low incidence of TCMR episodes that occurred in our study. As rejection episodes cannot be foreseen, the laborious sampling of prospectively included transplant recipients was performed at our center beginning at the day of transplantation. The vigorous sampling and inclusion of the majority of kidney transplant recipients made it possible to analyze six patients with a cellular rejection. To further overcome this shortcoming, we analyzed biopsy and blood samples of additional seven kidney transplant recipients from a historical cohort and could confirm our findings.

A specific strength of this paper is the closed prospective cohort study design and homogenous patient population with high internal validity. All patients received the same non-lymphodepletional immunosuppressive induction regimen. The prospective sampling allowed to draw conclusions on the T-cell-driven alloresponse at the timepoint of rejection. This made it possible to analyze the infiltrating T-cells, and by knowing the donor-reactive TCR repertoire and their CDR3 sequences, we were able to evaluate potential connections between clonotypes found in the tissue in a network-based approach.

Based on our data, we conclude that the donor-reactive T-cells of kidney transplant recipients also after non-lymphodepletional induction therapy increase in the peripheral blood stream after transplantation. We observed an accumulation of donor-reactive T-cells with substantially diverse TCRs, suggesting an activation of a broad number of T-cells directed against a variety of epitopes in the allograft, and the composition was not reflected by the peripheral blood stream. This is underlined by network analysis where predominant clusters of graft-infiltrating T-cells potentially directed toward a predominant antigen were not observed either in the circulating or in the graft-infiltrating T-cells. Nevertheless, we observed an importance of donor-reactive clonotypes in the allograft of kidneys experiencing a TCMR based on the CDR3 region, as they were found to be among the most centrally connected clonotypes despite their low frequency.

## Data Availability Statement

The datasets for this study can be found in the European Genome-phenome Archive (EGA, ID: EGAD00001007695).

## Ethics Statement

Institutional ethics committee approval (EC NB: 1973/2017, signed 7/11/2017; EC NB.: 20303-0/2010-1018EKU (821/PI/010)) from the Ethics Committee of the Medical University of Vienna was obtained for all aspects of the study and all study participants were included after signed informed consent prior to transplantation.

## Author Contributions

RO, RR-S, and JW were responsible for the conception, design, financial support, critical revision, and final approval of the manuscript. RO, CA, and AH were responsible for the manuscript writing. KJ, KH, AH, JW, LP, GG, ME, ART, HR, and CA performed the sample collection, *in vitro* experiments, and data analysis. TW, MS, JBH, RR-S, HR, and KJ are responsible for the critical revision of the manuscript. AH, MG, SS, SW, JV, and AK are responsible for the statistics, bioinformatic analysis, and revision of the manuscript. All authors read and approved the final manuscript. All authors contributed to the article and approved the submitted version.

## Funding

The study was founded by a peer-reviewed funding by the Scientific Funds of the Austrian National Bank-OeNb project number 17289 (https://www.oenb.at). The funding body had no influence on the design, collection, analysis, and interpretation of data and writing the manuscript.

## Conflict of Interest

The authors declare that the research was conducted in the absence of any commercial or financial relationships that could be construed as a potential conflict of interest.

## Publisher’s Note

All claims expressed in this article are solely those of the authors and do not necessarily represent those of their affiliated organizations, or those of the publisher, the editors and the reviewers. Any product that may be evaluated in this article, or claim that may be made by its manufacturer, is not guaranteed or endorsed by the publisher.
