# Supplementary material for: Prospective Tracking of Donor-Reactive T-Cell Clones in the Circulation and Rejecting Human Kidney Allografts
Source: Front Immunol. 2021 Oct 14;12:750005. doi: 10.3389/fimmu.2021.750005 (PMC8552542; doi:10.3389/fimmu.2021.750005)
Supplement: Supplementary file 1 [file DataSheet_1.pdf]

Supplementary Material

Supplementary table 1 Patients characteristics of historical cohort used for TCR beta sequencing of kidney biopsies

Subject	Gender	Cause of ESRD	MM-<br>%<br>PRA	Type of Tx	Rejection	Timepoint of Biopsy	Immunosuppressive Therapy
P03	Female	Unknown	3<br>0%	DKD	BANFF1	POD 46	Steroid. MMF. Tac
P06	Female	Hypertension	3<br>0%	DKD	BANFF1	POD 4	Steroid. MMF. Tac
P08	Male	Unknown	5<br>0%	DKD	BANFF1	POD 7	Steroid. MMF. Tac
P14	Female	GN	4<br>0%	DKD	BANFF1	POD 7	Steroid. MMF. Tac
P17	Male	Typ II Diabetes	3<br>0%	DKD	BANFF2	POD 3	Steroid. MMF. Tac
P19	Male	Unknown	5<br>26%	DKD	BANFF1	POD 8	ATG<br>+Steroid. MMF. Tac
P20	Female	Goodpasture Syndrome	2<br>0%	DKD	BANFF1	POD 4	Steroid. MMF. Tac

POD: Postoperative day. MMF: Mycophenolatmofetil. MM: Sum of HLA mismatch (A-B-DR). DKD: Diseased kidney donation. ATG: Anti-thymocyte globulin. GN: Glomerulonephritis

Supplementary table 2 Pre-transplant unique clonotype and clone counts for each subject divided in CD4 and CD8 T-cells

Patient	Group	CD4 clonotypes	CD4 copies	CD8 clonotypes	CD8 copies	Total clonotypes	Total copies
R14	Control	177989	317499	15055	51873	193044	369372
R148	Control	132197	352544	43325	183194	175522	535738
R190	Control	40743	59127	87736	212804	128479	271931
R24	Control	60960	227966	7389	170925	68349	398891
R30	Control	107755	327470	11068	68017	118823	395487
R32	Control	128623	333378	71747	258484	200370	591862
R106	Rejection	15830	36418	3477	38761	19307	75179
R132	Rejection	181478	672855	15884	370839	197362	1043694
R172	Rejection	100954	195294	31353	183112	132307	378406
R34	Rejection	118686	281430	77176	192893	195862	474323
R42	Rejection	78310	127739	28047	54117	106357	181856
R43	Rejection	187968	426701	141843	625776	329811	1052477

Supplementary table 3 MLR unique clonotype and clone counts for each subject divided in CD4 and CD8 T-cells

Patient	Group	CD4 clonotypes	CD4 copies	CD8 clonotypes	CD8 copies	Total clonotypes	Total copies
R14	Control	74755	1261207	4518	284886	79273	1546093
R148	Control	76302	1009450	53308	1147196	129610	2156646
R190	Control	76305	878426	28738	981699	105043	1860125
R24	Control	12516	106759	2739	50614	15255	157373
R30	Control	156740	815510	2113	75780	158853	891290
R32	Control	61264	720468	9126	713551	70390	1434019
R106	Rejection	16507	27395	2629	73144	19136	100539
R132	Rejection	170208	1735366	14989	754603	185197	2489969
R172	Rejection	79442	1110709	1440	77671	80882	1188380
R34	Rejection	11904	353252	2791	12479	14695	365731
R42	Rejection	68855	1076096	45446	1321632	114301	2397728
R43	Rejection	228435	1977466	162544	2072008	390979	4049474

Supplementary table 4 Post transplantation unique clonotype and clone counts for each subject divided in CD4 and CD8 T-cells

Patient	Group	CD4 clonotypes	CD4 copies	CD8 clonotypes	CD8 copies	Total clonotypes	Total copies
R14	Control	84538	151624	21703	82835	106241	234459
R148	Control	164173	540998	78415	418690	242588	959688
R190	Control	148179	302123	107762	260352	255941	562475
R24	Control	83306	335402	8768	280565	92074	615967
R30	Control	36983	63973	8678	27215	45661	91188
R32	Control	138311	447242	20336	71109	158647	518351
R106	Rejection	62045	116791	7000	28078	69045	144869
R132	Rejection	151307	519571	7730	154531	159037	674102
R172	Rejection	166483	499757	78028	504458	244511	1004215
R34	Rejection	117804	248889	20867	33911	138671	282800
R42	Rejection	110626	209464	29975	62186	140601	271650
R43	Rejection	181714	433749	89411	308574	271125	742323

Supplementary table 5 Top 20 clonotypes found in tissue allograft in subject R34 and R43 ordered by frequency

R34	cdr3nt	tissue-rank	tissue-freq	periph-rank	periph-freq
	TGTGCCAGCAGCCAGGCCTCGGAACAGCAGTACTTC	1	0.012124151	222	0.000140694
	TGCGCCAGATCAGCGGGACTGGACAATGAGCAGTTCTTC	2	0.00937601		
	TGCAGTGCTAGAGGGCTAGCGGGAGACCAAACCAAAGAGACCCAGTACTTC	3	0.006142903	5	0.001680058
	TGCGCCAGCAGCTCCGGGACAGCCCATCCCTACGAGCAGTACTTC	4	0.004364694	78	0.000264837
	TGTGCCAGCAGCTCCGTAGCGGGAGGGCCCGAGAATGAGCAGTTCTTC	5	0.004203039	12	0.000819338
	TGTGCCAGCAGCTTGGCCGGGACACACAATGAGCAGTTCTTC	6	0.004041384	312	0.00010759
	TGTGCCAGCAGTAAAGCCCAGGGGGGACAAGAGACCCAGTACTTC	8	0.003718073	106	0.000206904
	TGCGCCAGCAGCCAAGCCAGAACAGGGGGGCTCGGCTACACCTTC	8	0.003718073	1043	4.96569E-05
	TGTGCCAGCAGCCTTGGCCCGGCCCCCTACGAGCAGTACTTC	9	0.003556418	2123	3.16771E-05
	TGTGCCTGGAGTTTTGTCGTCCCCCCACGGGAGCTGTTTTTT	10	0.003394762	32	0.000413807
	TGTGCCATCGGGTTACTGGACGGGACTTCTGGGGCCAACGTCCTGACTTTC	11	0.003233107	3	0.003062174
	TGTGCCAGCAGCTTAATGTGGGGTGTTTACGGAAACACCATATATTTT	12	0.003071452	1043	4.96569E-05
	TGCGCCAGCAGCCCCGACGCCTGGAACAATCAGCCCCAGCATTTT	14	0.002909796		
	TGTGCCAGCCGCCCCGCGTACAATGAGCAGTTCTTC	14	0.002909796	164	0.000165523
	TGTGCCAGCAGCTTAGGGACTAGCGTCTACGAGCAGTACTTC	15	0.002748141	16633	1.15189E-05
	TGTGCCAGCAGCTTAGCGGGGTCCTACAATGAGCAGTTCTTC	17	0.002586486	312	0.00010759
	TGTGCCAGCAGCGCAAGCGGGACCTACGAGCAGTACTTC	17	0.002586486		
	TGTGCCAGCGGGGGACAGTGGGCCGGCTACACCTTC	18	0.00242483		
	TGCAGCGTTGAAAGTGGGTATCGCTCTGAAGCTTTCTTT	19	0.002263175	552	7.44853E-05
	TGTGCCAGCAGTCCACTAGGCCCCTACAAGACAGGATACACCGGGGAGCTGTTTTTT	22	0.00210152	164	0.000165523

R43	cdr3nt	tissue-rank	tissue-freq	periph-rank	periph-freq
	TGTGCCAGCAGTGTGACTAGCGGCCCCTACAATGAGCAGTTCTTC	1	0.013198892	5	0.005745543
	TGTGCCAGCAGCTTAGCCCAGGGACCCATCGTTGGGTACAATGAGCAGTTCTTC	2	0.006967284	1	0.063858097
	TGTGCCAGCAGCTTAACGGGACTAGTTAATCAAGAGACCCAGTACTTC	3	0.005755582	2	0.033833359
	TGTGCCAGCAGTCCTAGGGGCACCGGGGAGCTGTTTTTT	4	0.004500606	285	8.24484E-05
	TGTGCCAGCAGCCAAGAACTAGCGGGAGGCCCAGATACGCAGTATTTT	5	0.00402458	37	0.000428239
	TGTGCCAGCAGTTTATTTGGGGCTAGCGGGAGAGGTGAGCAGTTCTTC	6	0.00367838	16	0.001596052
	TGCGCCAGCAGGGGGGAGGGCTTGCATGAGCAGTTCTTC	7	0.002553228	50	0.000337177
	TGTGCCAGCAGCTTATCGGGCTCCTACGAGCAGTACTTC	8	0.002509953		
	TGCGCCAGCATAAGCTCTGGGTTGACAGATACGCAGTATTTT	9	0.002423403		
	TGCGCCAGCAGCCTCCAGGGCGTCGGACAGGGGAACACCGGGGAGCTGTTTTTT	10	0.002250303		
	TGTGCCAGCAGCCCCAACGAAGAGCGGGATTACTACAATGAGCAGTTCTTC	12	0.002207028	20	0.001080443
	TGTGCCAGCAGTTCCCCAGGGTATAGCGACATTCAGTACTTC	12	0.002207028	1206	2.83032E-05
	TGTGCCAGCAGCTCTAGCGGGAGCGTCCAAGAGACCCAGTACTTC	13	0.002163753		
	TGTGCCAGCAGCTTATCCCAGGCAGATACGCAGTATTTT	14	0.002120478	10	0.004394374
	TGTGCCAGCAGCTTAGCCATTCTGGGTTACGAGCAGTACTTC	15	0.002033928	293	7.99872E-05
	TGTGCCAGCAGCCGCACGAGCGGGCTTAAGGCAGATACGCAGTATTTT	16	0.001947378	337	7.13732E-05
	TGCGCCAGCAGTGGGGCTAGCGGGACCACCGGGGAGCTGTTTTTT	17	0.001860827	11540	7.38343E-06
	TGCGCCAGCAGCCGAACCGACAGAGTAAAAACCGGGGAGCTGTTTTTT	18	0.001774277		
	TGTGCCAGCAGCCCGTTCCCCGGACAGCGCTCCTACAATGAGCAGTTCTTC	19	0.001601177	326	7.38343E-05
	TGCGCCAGCAGCTTGGACAGCTCCTACGAGCAGTACTTC	20	0.001514627		

Supplementary table 6 Jensen-Shannon Divergence (JSD) of VJ usage of the circulating and graft infiltrating TCR repertoire. The low JSD values when comparing the peripheral blood and allograft indicates a similar VJ usage in these two compartments at the same timepoint.

Patient	*Variable*	*Primer*	*JSD*
P03	PostTx vs Tissue	TRBA	0.1367
P03	PostTx vs Tissue	TRBB	0.1870
P06	PostTx vs Tissue	TRBA	0.1121
P06	PostTx vs Tissue	TRBB	0.0804
P08	PostTx vs Tissue	TRBA	0.0775
P08	PostTx vs Tissue	TRBB	0.0268
P16	PostTx vs Tissue	TRBA	0.0501
P16	PostTx vs Tissue	TRBB	0.0389
P18	PostTx vs Tissue	TRBA	0.1091
P18	PostTx vs Tissue	TRBB	0.0657
P20	PostTx vs Tissue	TRBA	0.2125
P20	PostTx vs Tissue	TRBB	0.0735
P21	PostTx vs Tissue	TRBA	0.1287
P21	PostTx vs Tissue	TRBB	0.1160

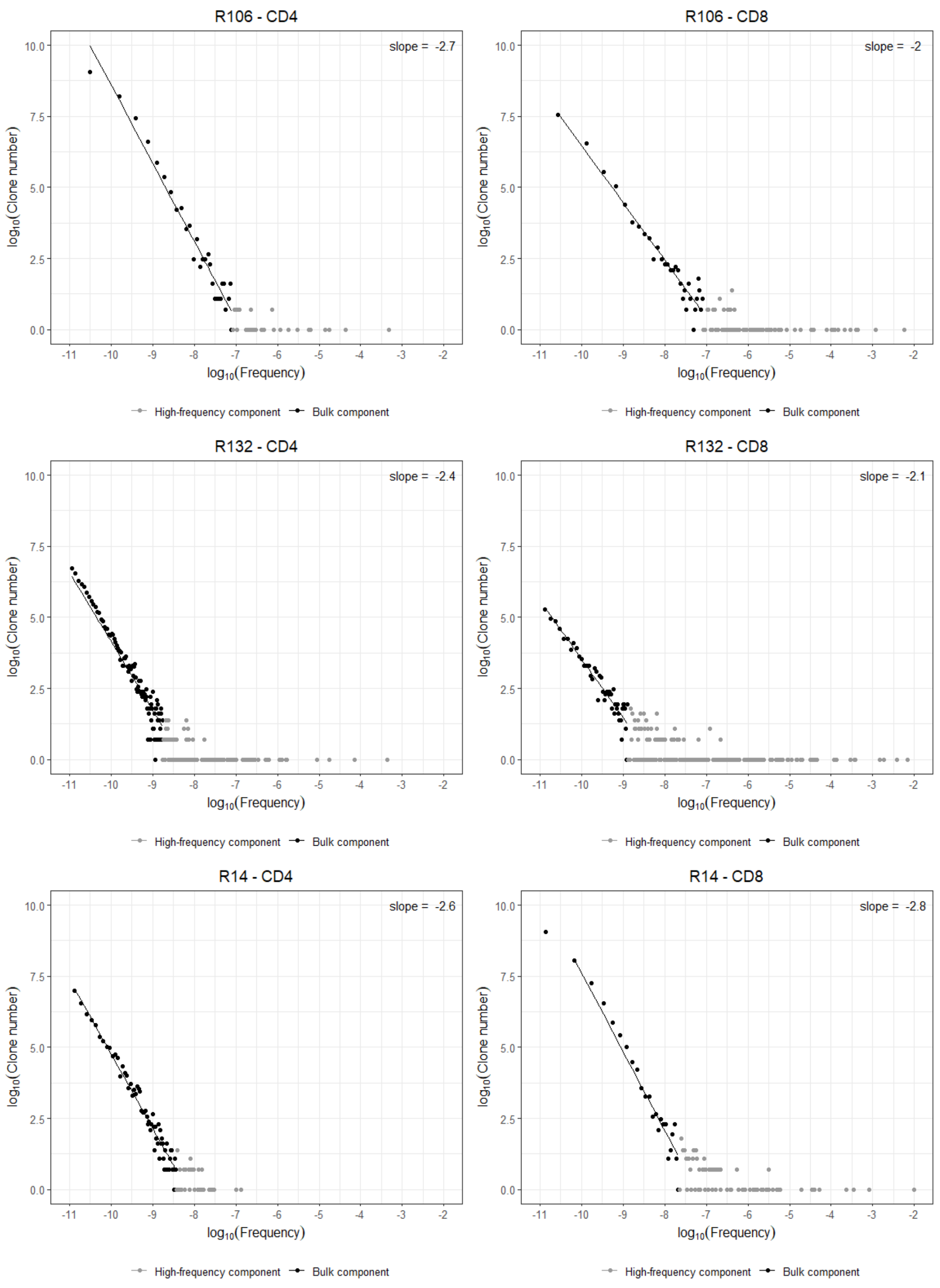

Supplementary figure 1 Power law slopes of the bulk component of selected unstimulated repertoire samples grouped by phenotype.

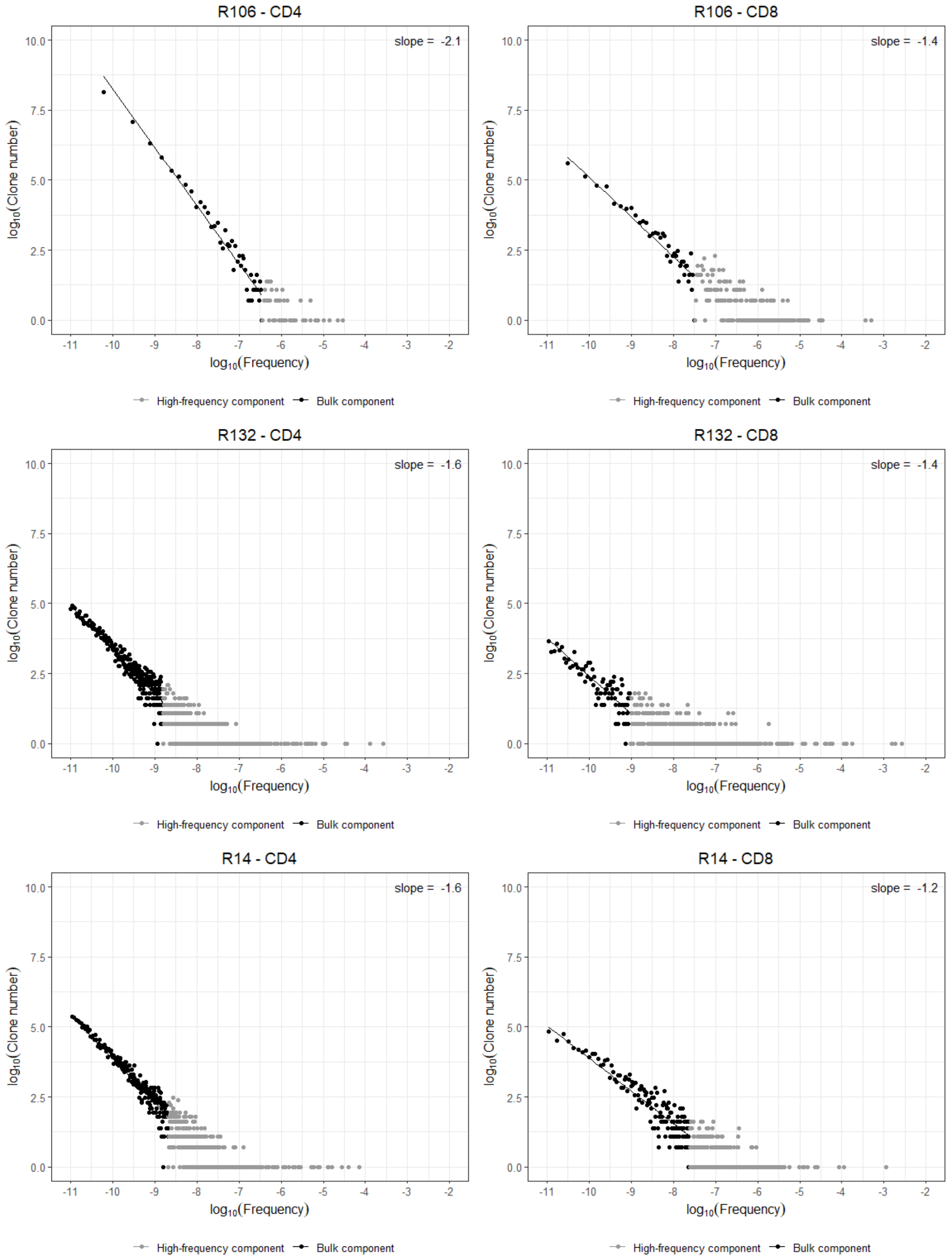

Supplementary figure 2 Power law slopes of the bulk component of selected donor-reactive repertoire samples grouped by phenotype

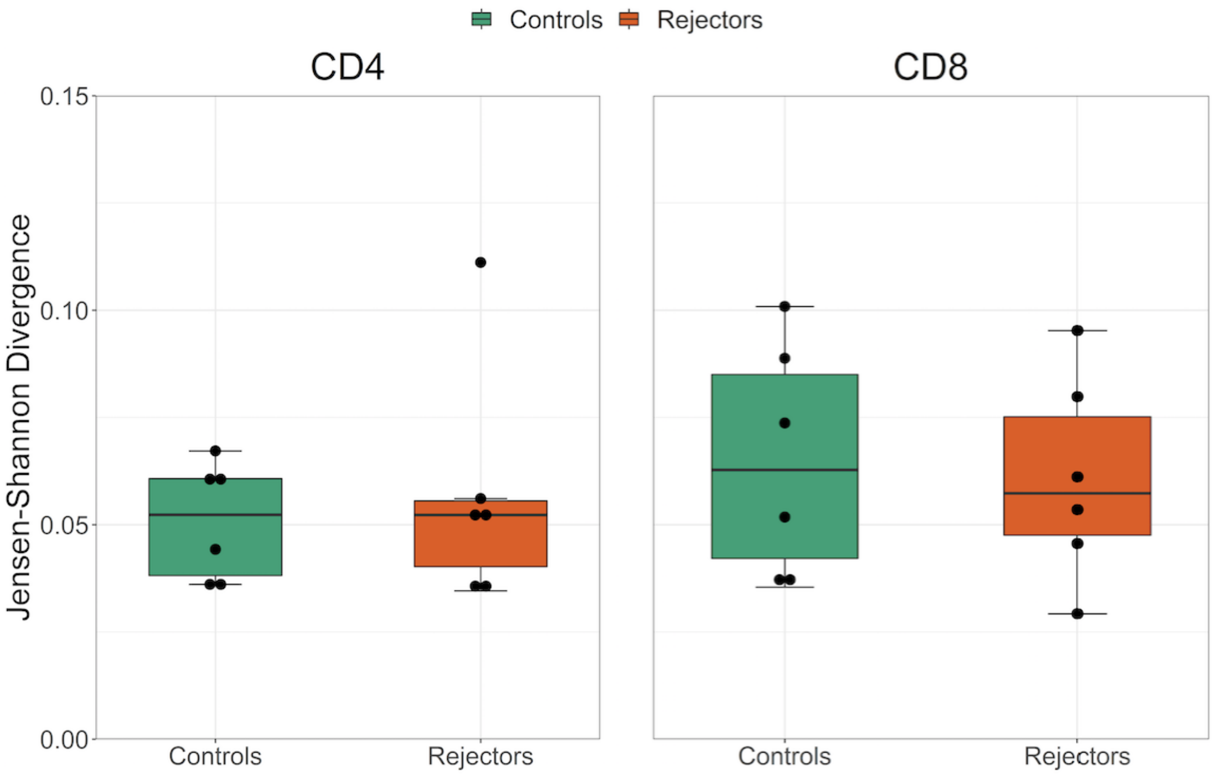

Supplementary figure 3 Boxplot of the Jensen-Shannon divergence (JSD) of the top 1000 clones between the pre-transplant and post-transplant samples grouped by the outcome T-cell mediated graft rejection

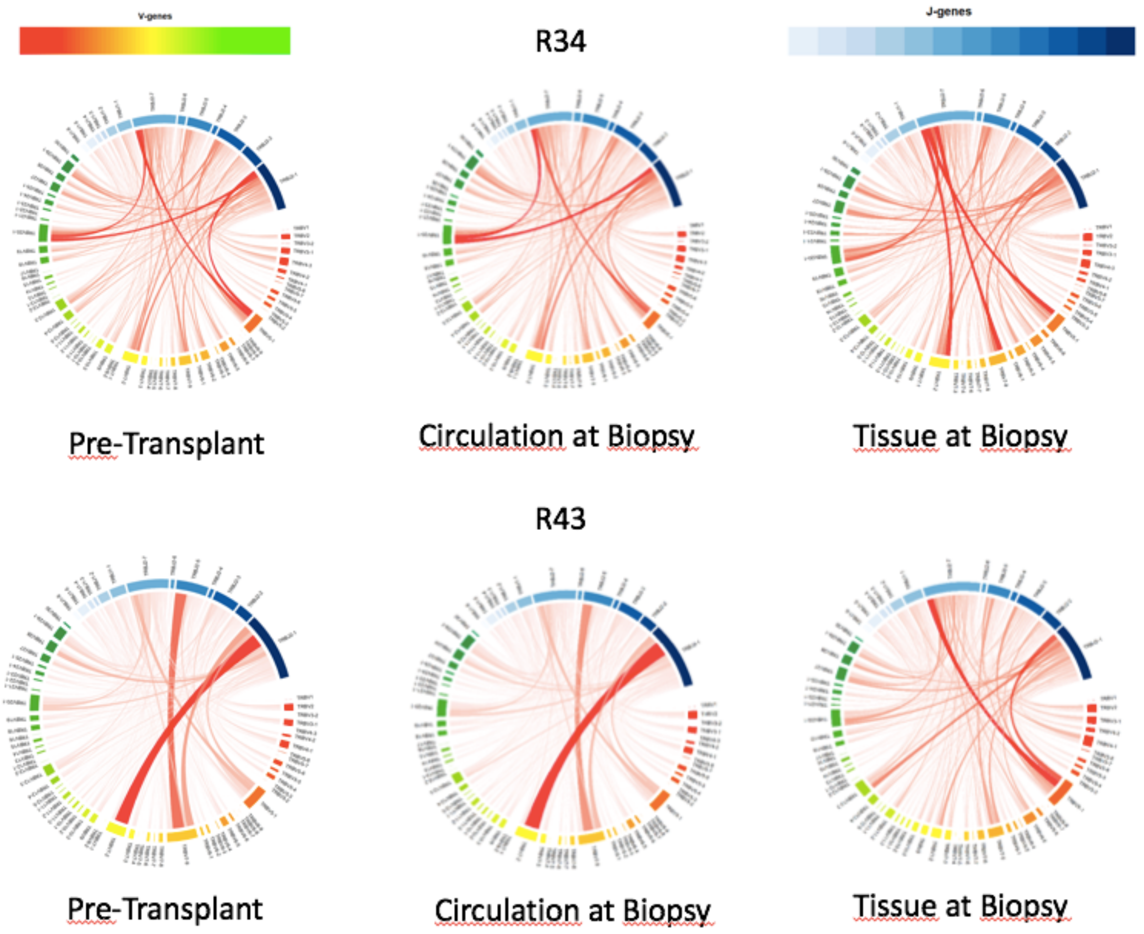

Supplementary figure 4 Graphical comparison of VJ combinations in the Circulation and the allograft at biopsy of 2 rejecting patients. The overall V-J usage in periphery and tissue remained the same between the circulation and allograft and JSD for VJ usage between in the circulation and tissue were 0.065 for patient R34 and 0.063 patient R43.

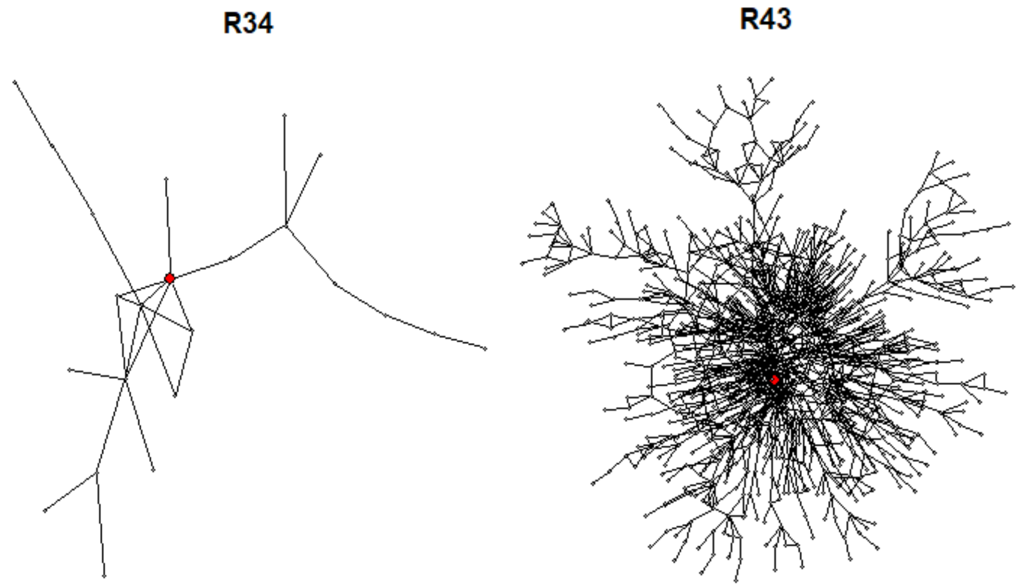

Supplementary figure 5 Largest cluster of the subject-specific tissue networks of R34 and R43. The node with the highest betweenness centrality is represented as a red node.
